# Editorial: Novel insights into CAR T-cell associated neurotoxicity, volume II

**DOI:** 10.3389/fneur.2026.1885869

**Published:** 2026-06-29

**Authors:** Stella Bouziana, Piers E. M. Patten, Thomas Skripuletz, Nora Möhn, John De Groot

**Affiliations:** 1 Department of Haematology, King's College Hospital NHS Foundation Trust, London, United Kingdom; 2 Comprehensive Cancer Centre, King's College London, London, United Kingdom; 3 Department of Neurology, Hannover Medical School, Hannover, Germany; 4 Center for Neurology, Department of Neuroimmunology and Neuromuscular Diseases, University Hospital and University Bonn, Bonn, Germany; 5 Department of Neurological Surgery, University of California, San Fracisco, San Francisco, CA, United States

**Keywords:** CAR T-cells, hematological malignancies, ICANS, neurotoxicity, solid tumors

The landscape of chimeric antigen receptor (CAR) T-cell therapy continues to evolve at a remarkable pace. Since the first FDA approval in 2017, the therapeutic indications for CAR T-cell products have expanded considerably across hematological neoplasms and beyond malignancies. This rapid application of CAR T-cell therapies stipulates the need of deepening the understanding, detection and management of CAR T-cell driven toxicities such as neurological complications. Immune effector cell-associated neurotoxicity syndrome (ICANS) remains the most clinically significant neurological complication with the potential to evolve to a life-threatening condition. Clinical manifestations encompass a range of presentations, including aphasia, altered consciousness, cognitive impairment, seizures, and in severe cases cerebral oedema (1). According to a meta-analysis of 75 trials across hematologic malignancies, ICANS occurs in approximately 27% of treated patients across all grades and 10.5% at high grade, with marked variation across CAR constructs and target antigens (2).

Beyond the classical forms of ICANS, accumulating evidence has revealed distinct and rare late-onset neurotoxicity syndromes which can emerge particularly in patients receiving B-cell maturation antigen (BCMA)-directed CAR T-cell therapies such as ciltacabtagene autoleucel. These rare syndromes comprise movement and neurocognitive treatment-emergent adverse events, peripheral nerve palsies, and parkinsonian features carrying significant morbidity and incurring fatal outcomes at high rates (3). In addition, there are reports of ischemic neurological episodes and myelopathy mostly described after anti-CD19 CAR T-cell therapy (4). The mechanistic basis of late neurotoxicity is not fully elucidated. On-target, but off-tumor toxicity mediated by CAR T-cells recognizing BCMA expressed on cells of the basal ganglia has been proposed. At the same time, central pathways to ICANS such as cytokine-mediated endothelial activation and blood–brain barrier (BBB) disruption may co-exist and contribute to the delayed phenotype (5).

Understanding of ICANS pathogenesis has been substantially refined and it seems to be multifactorial, involving cytokine-mediated endothelial activation, disruption of the BBB, glial activation, neurovascular uncoupling and potential direct on-target off-tumor toxicity mediated by CD19 expression on cerebrovascular pericytes in the context of anti-CD19 therapies (6). Myeloid-derived interleukin (IL)-1 and IL-6 production and endothelial biomarkers, such as angiopoietin-2 (Ang-2), von Willebrand factor and Ang-1, have been associated with ICANS severity (7).

Despite considerable advances in mechanistic understanding, clinical assessment remains challenging, treatment is primarily glucocorticoid based, and the differential diagnosis from other encephalopathies including infectious etiologies, remain significant clinical burdens. To successfully address these challenges, specialists across different fields should join forces to advance effective management approaches.

The first volume of this Research Topic provided a comprehensive overview of CAR T-cell neurotoxicity, from pathophysiological mechanisms to emerging therapeutic approaches (8). This second volume aimed to collect emerging articles which address three areas of pressing unmet need; earlier and more sensitive detection of neurotoxicity, optimization of treatment in refractory cases, and the critical importance of rigorous differential diagnosis. All submitted articles in this Research Topic underwent a rigorous peer review process and finally five articles were published.

1. In their review, Yamamoto et al. characterize immunotherapy-associated dysgraphia as an early and under-recognized neurocognitive manifestation of ICANS in patients treated with CAR T-cells for hematological malignancies. The authors synthesize clinical evidence demonstrating that handwriting impairment, although already incorporated as a domain within the immune effector cell encephalopathy (ICE) score, may reflect deeper neurocognitive dysfunction when assessed in greater detail. The authors discuss the mechanistic basis and practical challenges of standardized dysgraphia assessment in the clinical setting.

2. Aziz et al. present a comprehensive review of ICANS pathophysiology, integrating cytokine-mediated endothelial injury, BBB disruption, glial activation and neuroinflammation into a unified mechanistic framework. The authors propose a structured classification of predictive biomarkers across pre-infusion, early on-treatment, and post-treatment timepoints, and explore translational strategies for ICANS prevention. Computational and predictive modeling approaches are discussed as emerging promising tools for improving predictive power while maintaining biological interpretability, while implementation challenges are presented.

3. Völker et al. report the development and prospective bicentric validation of the Berlin-Hannover ICANS severity assessment (BHISA), a novel bedside tool designed to address the sensitivity limitations of the ICE score. By incorporating additional cognitive and motor domains, the BHISA demonstrated superior performance in detecting early and subtle neurocognitive changes mainly in patients with hematological malignancies treated with commercial or in-house produced CAR T-cells, while maintaining clinical feasibility at the bedside. The authors advocate for prospective multicentre validation to refine screening strategies and support more personalized management of CAR T cell-associated neurotoxicity.

4. In a retrospective single-center study, Meng et al. report early intrathecal dexamethasone and methotrexate (IDM) administration in 13 patients with hematological malignancies who developed severe or steroid-refractory ICANS. An overall response rate of 92.3% was observed with rapid resolution of symptoms and concomitant reductions in CAR T-cell counts and IL-6 levels in cerebrospinal fluid. These findings place IDM as a promising strategy for refractory ICANS with a direct intrathecal mechanism bypassing the limitations of systemic immunosuppression.

5. Birk et al. present a case report of severe human herpesvirus 6 (HHV-6)-related encephalitis in an adult patient with refractory transformed follicular lymphoma treated with an academic autologous 4-1BB anti-CD19 CAR-T product as part of a clinical trial. Interestingly, no evidence of HHV-6 was found within the apheresis product, transduced CAR T-cells, and *in vitro* activated CAR T-cells, implicating host immune reconstitution dynamics rather than CAR T cell-mediated viral amplification. The case illustrates the diagnostic complexity of differentiating viral encephalitis from ICANS, particularly given overlapping clinical features and atypical steroid response, and underscores the need for systematic viral screening in patients with neurotoxicity not responding as expected to standard management.

The contributions in this volume highlight that progress in managing CAR T cell-related neurotoxicity requires advances across detection, unraveling pathophysiological mechanisms, introducing preventing strategies, and progress in treatment approaches and differential diagnosis. As indications for CAR T-cell therapies continue to broaden, we hope that the second volume of this Research Topic raises scientific questions and provokes future work in this rapidly evolving field.
